# Maximum phonation time in pre-school children

**DOI:** 10.1016/S1808-8694(15)30602-9

**Published:** 2015-10-18

**Authors:** Carla Aparecida Cielo, Viviane Michele Cappellari

**Affiliations:** 1 Doctor, professor of the Otorhino-Phonoaudiology Department and of the post-graduate program on Human Communication Disorders, Universidade Federal de Santa Maria/RS.; 2 Master in Human Communication Disorders - UFSM. Speech therapist. Universidade Federal de Santa Maria.

**Keywords:** acoustic analysis, children, speech therapy, maximum phonation time, voice

## Abstract

Past studies on the maximum phonation time (MPT) in children have shown different results in duration. This factor may reflect the neuromuscular and aerodynamic control of phonation in patients; such control might be used as an indicator of other evaluation methods on a qualitative and quantitative basis.

**Aim:**

to verify measures of MPT and voice acoustic characteristics in 23 children aged four to six year and eight months.

**Method:**

The sampling process comprised a questionnaire that was sent to parents, followed by auditory screening and a voice perceptive-auditory assessment based on the R.A.S.A.T. scale. Data collection included the MPT.

**Study:**

a prospective and cross-sectional study.

**Results:**

The MPT was 7.42s, 6.35s and 7.19s; as age increased, the MPT also increase significantly; the s/z relation at all ages was close to one.

**Conclusions:**

The results mostly agree with the medical literature. MPT values, however, were higher than in other Brazilian studies. It may be concluded that the ages that were analyzed are going through neuromuscular maturation; lack of structural maturity and neuromuscular control was more evident in chidlren aged four years.

## INTRODUCTION

An assessment is the starting point for the diagnosis and treatment of any voice disease or for training one's voice. The importance of an adequate evaluation continues throughout voice treatment/voice training as a form of measuring possible progression of the patient/client and in defining the adequate moment for ending clinical treatment.

Considering the speed at which organic and physiological change takes place in children, it is essential for voice professionals (speech therapists, otorhinolaryngologists or singing teachers) to monitor the progression of their patients/clients to adapt their approach towards improved results.

Various studies to establish normalcy levels for the indices used in voice assessment have been undertaken in the context of voice studies. These studies have included establishing the maximum phonation time (MPT), given that this measurement is one of the most common indicators used in clinical voice evaluation; it is easily measured and is considered an objective acoustic measurement of glottic efficiency.

Brazilian and international studies on the MPT in children have reached conflicting results. All of them, however, have shown that the MPT is a measurement that may reflect neuromuscular and aerodynamic control of voice in any individual. Thus, speech therapists, otorhinolaryngologists and singing teachers may use the MPT as pre-evaluation for other qualitative or quantitative assessments.

This study did not aim to establish normalcy parameters, but rather, to help verify MPT measurements in children aged four to six years, of both sexes, studying in public and private preschools.

## MATERIAL AND METHOD

### Bioethical issues

The initial contact was made with four public and private preschools that agreed to participate in the study; an institutional authorization term was used to elect the best method for explaining the study to parents and caretakers. Parents or caretakers that agreed to allow their children to participate in the study signed a free informed consent form. The institutional term and the consent form were based on the guideline 196/96 (CONEP) and were approved by the Research Ethics Committee (protocol number 099/05.

### Description of the sample

The population from which the sample was taken consisted of children of both sexes, aged four to six years and eight months, who studied in public and private preschools of a major city. Inclusion criteria were: absence of airway infection at the time of the evaluation; auditory thresholds within the normal range; no history of neurological, psychiatric or gastric diseases; children that did not sing in choirs and/or as soloists; absence of previous phonoaudiological therapy; no past laryngeal surgery; and voice considered as adequate or normal. These criteria resulted in 23 subjects.

### Description of the material

The following material was used in the sample and data selection process: a questionnaire based on the literature, an Amplivox model A260 audiometer, a TK Missouri otoscope, a Casio chronometer, a Creative model Muvo TX FM USB 2.0 256MB memory coupled microphone digital recorder, and a Wave Pad v. 3.05 NHC Swift Sound audio editing software running on a PC Pentium 266HHZ CD-ROM 16 MB RAM computer.

### Procedures

Sample selection procedures

The selection procedure assessed 104 children; their parents or caretakers answered a questionnaire about the general health status of each child, including questions about past neurological, psychiatric or gastric disease, whether the children sang in choirs and/or as soloists, whether they had undergone phonoaudiological therapy or laryngeal surgery.^1^

Children also underwent an auditory evaluation according to standards described in the literature^2^ in a quiet room, done by a speech therapist. This evaluation consisted of screening for speech frequencies (1000 and 2000 Hz at 20 dBNA, and 4000 Hz at 25 dBNA)^2^ and the results were annotated in a customized protocol, which was sent to the next screening phase. This second phase consisted of perceptive and auditory evaluation of voice for subjects with auditory thresholds equal to or below 20 dB at 1000 and 2000 Hz and equal or below 25 dB at 4000 Hz, which indicated normal hearing in both ears.

Eighty-three of 104 children were approved in this selection phase; these children then underwent the auditory-perceptual evaluation of voice as the last step in the sample selection process.

Voice samples were not taken from five subjects (of 83 subjects) who were not present at school during the selection period. The 78 selected subjects were taken out from class individually with approval by teachers, and sent to a silent and reserved room. Each subject was asked to stand with arms extended along the body; the digital recorder was placed 4 cm from the subject's mouth.^3^, ^4^, ^5^, ^6^ The subject was asked to breath in deeply and to issue the sustained phoneme /a/ at a typical (normal voice) speech height, intensity, quality and velocity.^3^, ^6^, ^7^, ^8^, ^9^

These voice samples were sent to four referees who were speech therapists with at least five years experience in voice evaluation, who did not know the purposes of the study, and who analyzed the voices independently, without knowing about the remaining referees.^12^

The auditory-perceptual evaluation of voice was based on the RASAT scale,^13^ which is a translation into Portuguese of the GRBAS scale developed by the Japanese Laryngology Society.^14^ The RASAT scale evaluates the grade of hoarseness (R), roughness (A), breathiness (S), asthenia (A) and strain (T); each parameter may be graded as 0 - normal, 1 - mild, 2 - moderate, and 3 - severe.

Based on methods in other studies, the result adequate or adapted voice (normalcy) was based on the mean of the four verdicts by the referees for each RASAT parameter.^12^, ^15^, ^16^, ^17^, ^18^, ^19^, ^20^, ^21^, ^22^ In this study, the normalcy criteria were a mean grade not over 0.9 in at least four of five RASAT parameters, or a mean grade not over 1 in one of five RASAT parameters.^12^

This evaluation was extremely important, as its results defined voices as normal or altered, and thus, whether any subject was included or not. After consultation with four otorhinolaryngologists, it became a consensus that indirect laryngoscopy was not feasible during the selection phase, given the age range and the number of children. These physicians recommended optic nasofibrolaryngoscopy as the best form of evaluating these children; this option was also discarded, given the difficulty in bringing in the whole group into medical offices. This explains the choice for a rigorous auditory-perceptual evaluation of voice, as described above, as a reliable and feasible method for identifying dysphonia and selecting the sample.

The auditory-perceptual evaluation of voice based on the RASAT scale excluded 55 children classified as dysphonic. There remained 23 subjects with adapted (normal) voices, which were selected as the sample for this study. Seven of these children were aged between 4 years and 4 years 11 months (n=7); eleven were aged between 5 years and 5 years 11 months (n=11); and five children were aged between 6 years and 6 years 8 months (n=5).

### Data collection procedures

While collecting the voice sample for the auditory-perceptual evaluation of voice (selection phase), the same voice sample was used for the MPT (data). Each subject was asked to breath in deeply and to issue the sustained phoneme /a/, /s/ and /z/ at a typical (normal voice) speech height, intensity, quality and velocity.^3^, ^6^, ^7^, ^8^, ^9^

All subjects issued the sustained phonemes until the end of expiration three times; a mean 10 second interval between each phoneme was given.^10^, ^11^ No child knew beforehand how many times they would be required to issue phonemes; this was done to avoid any bias on the results.^12^

A chronometer was used for measuring phonation times; MPT data was annotated in a specific protocol for tabulation. Subjects were grouped in tables into three age groups (four, five and six years). The minimum, maximum and mean values and the standard deviation for the sustained phonemes /a/, /s/ and /z/ was calculated for each age group. The same procedure was done considering the three age groups as a single group (full sample).

MPT measurements were analyzed and the results were normal or altered for each age group and for the full group. Normality was the mean of minimum and maximum values taken from other MPT measurement studies in children. There means have been established for each age group, as follows: age four years - 4 to 9.6 s; age five years - 5 to 9.36 s; age six years - 6 to 14.2 s.^4^, ^8^, ^15^, ^23^, ^24^, ^25^, ^26^, ^27^

MPT measurements below normal values were interpreted as suggesting transglottic air leak; MPT measurements above normal values were interpreted as suggesting increased glottic strain.^3^, ^4^, ^10^, ^27^

The /s/ and /z/ MPT ratio was noted; this measurement is obtained by dividing the /s/ MPT value by the /z/ MPT value. The normal interval for the s/z ratio in this study was obtained from data in the literature on the minimum and maximum values for children at the pertinent age groups. The mean minimum value thus obtained was 0.78 and the maximum value was 1.02. Value above this range were taken a suggesting air leaks during phonation, and values below this range were considered as suggesting increased glottic strain.^3^, ^4^, ^10^, ^15^, ^24^, ^27^

### Statistical interpretation

The statistical analysis was done using descriptive statistics (mean, median, standard deviation, minimum value and maximum value), Student's “t” test, Pearson's correlation coefficient, and the analysis of variance (ANOVA). A 5% (p ≤ 0.05) significance level was used in the conclusions. Findings were computed in the SPSS software, version 11.5.

Statistical significance was verified for the means at each age group, the total means, and the normality intervals. The sample was homogenized, notwithstanding the different number of subjects in each age group, as all of the analyses were based on the means per age group and for the full sample. Spearman's correlation analysis was applied to the results to verify the reliability of the referees's RASAT analysis.

## RESULTS

Mean MPT measurements for /a/ (p= 0.007), /s/ (p=0.0001) and /z/ (p=0.004) increased in parallel, as age increased. The /s/ and /z/ MPT measurements increased 2 s on average from one age group to the other. Pearson's correlation coefficient showed a positive statistical correlation between the mean of the full sample for the /a/ MPT and age (p=0.007); in other words, as the mean age increased, so did the /a/ MPT. There was also a positive correlation between the mean of the full sample for the /a/ MPT and the total means of the /s/ MPT (p=0.003) and the /z/ MPT (p=0.0001), showing that as the /a/ MPT increased, so did the /s/ and /z/ MPTs.

There was a statistically significant difference between /a/ MPT means at ages four and six years (p=0.024), the mean /a/ MPT at age six years was significantly higher than the mean /a/ MPT at age four years. There was also statistical significance in the comparison between the /a/ MPT mean at age six years and the full sample (p=0.022), showing that the /a/ MPT mean at age six years was significantly higher than the /a/ MPT mean of the full sample.

**Table 1:** Means of all parameters evaluated by age group and for the full sample

parameters	MEAN four years	MEAN five years	MEAN six years	TOTAL MEAN
/a/ MPT s	5,77	7,16	10,32	7,42
/s/ MPT s	4.73	6,35	8,62	6,35
/z/ MPT s	5,32	7,30	9,55	7,19
s/z ratio	1,05	0,98	0,91	0,99

There was a statistically significant difference between /s/ MPT means at ages four and six years (p=0.02) and between the five and six year means (p= 0.050). The /s/ MPT mean at age six years was significantly higher compared to the means at ages four and five years. There was also statistical significance (p=0.04) in the comparison between the /s/ MPT mean for the full sample and the mean at age four years; the full sample mean was significantly higher than the mean at age four years.

There was a statistically significant difference between /z/ MPT means at ages four and six years (p=0.025); the mean at age six years was significantly higher than the mean at age four years. There was, however, no statistically significant difference in the comparison between the /z/ MPT means between age groups and the full sample.

There was no statistically significant difference between age groups in the s/z ratio or in their comparison with the mean of the full sample.

The results were compared not only among the age groups, but also with data in the literature. As described above, a normal interval was defined. The means, minimum and maximum values, and the standard deviation per age group were also calculated, and are presented in Tables 2, 3, 4 and 5.

**Table 2:** Results per subject for /a/, /s/ and /z/ MPT measurements, in seconds, at age four years, compared to the literature

SN	Age **	Sex	/a/ MPT	Interval *	/s/ MPT	Interval *	/z/ MPT	Interval *
1	04:00	m	2,45	below	3,12	below	1,77	below
2	04:01	m	7,53	within	5,72	within	6,47	within
3	04:02	f	5,28	within	4,96	within	4,43	within
4	04:04	f	5,22	within	3,91	below	2,53	within
5	04:09	f	3,79	below	4,43	within	5,54	within
6	04:11	m	8,30	within	3,17	below	6,23	within
7	04:11	f	7,79	within	7,79	within	10,27	above
M			5,77	within	4,73	within	5,32	within
Mn			2,45		3,12		1,77	
Mm			8,30		7,79		10,27	
SD			2,20		1,64		2,82	

SN = subject number f = female m = male M = mean Mn= minimum Mm = maximum

SD = standard deviation

* Normal interval based on mean values of other studies for this age group

** The numeric representation in the column age refers to years:months

**Table 3:** Results per subject for /a/, /s/ and /z/ MPT measurements, in seconds, at age five years, compared to the literature

SN	Age **	Sex	/a/ MPT	Interval*	/s/ MPT	Interval*	/z/ MPT	Interval*
1	05:00	m	10,40	within	6,12	within	8,13	within
2	05:01	f	5,45	within	7,74	within	5,45	within
3	05:02	f	4,83	below	7,68	within	3,07	below
4	05:04	m	3,45	below	3,80	below	5,64	within
5	05:06	f	5,65	within	5,85	within	6,49	within
6	05:06	f	6,73	within	6,59	within	6,73	within
7	05:08	f	4,90	below	4,26	below	7,03	within
8	05:08	m	14,28	above	7,24	within	9,65	within
9	05:09	m	7,05	within	6,81	within	10,83	above
10	05:11	f	7,61	within	6,92	within	9,52	within
11	05:11	f	8,42	within	6,84	within	7,72	within
M			7,16	within	6,35	within	7,30	within
Mn			3,45		3,80		3,07	
Mm			14,28		7,74		10,83	
SD			3,04		1,28		2,21	

Normal interval based on mean values of other studies for this age group

SN = subject number f = female m = male M = mean Mn= minimum Mm = maximum

SD = standard deviation

** The numeric representation in the column age refers to years:months

**Table 4:** Results per subject for /a/, /s/ and /z/ MPT measurements, in seconds, at age six years, compared to the literature

SN	Age **	Sex	/a/ MPT	Interval*	/s/ MPT	Interval*	/z/ MPT	Interval*
1	06:01	m	9,02	within	8,33	within	7,46	within
2	06:01	m	11,12	within	6,74	within	9,94	within
3	06:02	m	12,90	within	9,21	within	11,28	within
4	06:04	m	10,16	within	12,06	within	12,06	within
5	06:08	m	8,39	within	6,76	within	7,03	within
M			10,32	within	8,62	within	9,55	within
Mn			8,39		6,74		7,03	
Mm			12,90		12,06		12,06	
SD			1,78		2,19		2,25	

Normal interval based on mean values of other studies for this age group

SN = subject number f = female m = male M = mean Mn= minimum Mm = maximum

SD = standard deviation

** The numeric representation in the column age refers to years:months

**Table 5:** Results per subject for the s/z ratio at ages four, five and six years, compared to the literature

	Subjects four years					Subjects five years					Subjects six years			
SN	Age **	Sex	s/z	Interval*	SN	Age **	Sex	s/z	Interval*	SN	Age **	Sex	s/z	Interval*
1	04:00	m	1,76	above	1	05:00	m	0,75	within	1	06:01	m	1,12	within
2	04:01	m	0,88	within	2	05:01	f	1,42	within	2	06:01	m	0,68	below
3	04:02	f	1,12	within	3	05:02	f	2,50	above	3	06:02	m	0,82	within
4	04:04	f	1,55	within	4	05:04	m	0,67	below	4	06:04	m	1,00	within
5	04:09	f	0,80	within	5	05:06	f	0,90	within	5	06:08	m	0,96	within
6	04:11	m	0,51	below	6	05:06	f	0,98	within					
7	04:11	f	0,76	within	7	05:08	m	0,75	within					
					8	05:08	f	0,61	below					
					9	05:09	m	0,63	below					
					10	05:11	f	0,73	within					
					11	05:11	f	0,89	within					
M			1,05	above	M			0,98	within	M			0,91	within
Mn			0,51		Mn			0,61		Mn			0,68	
Mm			1,76		Mm			2,50		Mm			1,12	
SD			0,45		DP			0,55		DP			0,17	

Normal interval based on mean values of other studies for this age group

Address for correspondence: Avenida Azenha 305/22 Bairro Azenha Porto Alegre RS 90160-000.

SN = subject number f = female m = male M = mean Mn= minimum Mm = maximum

SD = standard deviation

** The numeric representation in the column age refers to years:months

Comparing the results with data in the literature at ages four to six years showed that the means in this study are within normal limits, although the /a/, /s/ and /z/ MPTs of some subjects were below or above those normal limits.

The comparison of s/z ratio results with the literature at the ages of four to six years showed that only the mean at age four years was slightly above the normal limits described in the literature.

## DISCUSSION

The MPT is an acoustic measurement of voice and is widely used in the clinical setting for assessing voice function. It is commonly used by speech therapists, otorhinolaryngologists and singing teachers. Given its applicability, many studies have attempted to establish MPT measurement standards in children.

Various authors have stated that normal voice school age children should generally be capable of sustaining a sound for about ten seconds. It is, however, important to analyze our results by age group to attain more reliable and practical comparisons for use in voice clinics.^26^

The mean sustained /a/, /s/ and /z/ MPT per age group in this study increased with age, although a significant positive age correlation was only found for the /a/ MPT.

These results corroborate another paper^11^ that showed a similar increase in the MPT with age. Many studies on the MPT in children have been done on more extensive age groups and mean ages higher than those of our sample; thus, critical analyses are not possible.^11^, ^15^, ^28^ Few authors have investigated the MPT in children at ages that were similar to our sample and with means per age, which makes it possible to establish comparisons.

The mean /a/ MPT measurements in our results at ages four to six years were on average 3 s lower compared to the results in other papers.^23^, ^25^ At age six years, the difference was closer to 4 s.

The mean /z/ MPT measurement could only be compared at age five years, since the literature does not contain studies for this measurement at other ages. Our results were different by less than 1s compared to the results of another study.^24^

Brazilian authors have stated that children have mean MPT measurements close to their chronological age; for example, at age five years, a child would be able to sustain a sound for five seconds.4 In our study, subjects sustained the vowel /a/ and the fricative /s/ and /z/ sounds on average 2 s longer than their chronological age. This was observed even at age four years, when in theory there is less neuromuscular control.

The statistical analysis showed that children aged six years had significantly higher sustained /a/, /s/ and /z/ MPT measurements compared to children aged four years (/a/, /s/ and /z/ MPT at age six years > /a/, /s/ and /z/ MPT at age four years). Furthermore, a comparison between the means at each age group and the mean of the full sample showed that the mean was higher for the sustained /a/ in children aged six years compared to the mean of the full sample (/a/ MPT at age six years > /a/ MPT of the full sample). The difference between means was about 3 s higher at age six years compared to the full sample.

A similar comparison for the sustained fricatives showed that the /s/ MPT at age four years was significantly lower compared to the mean of the full sample (/s/ MPT at age four years < /s/ MPT of the full sample).

These data show that younger children may have normal MPT measurements for sustained /a/ and /z/, which are phonemes that occur by vocal fold vibration. On the other hand, their /s/ MPT measurements (in which vocal folds do not vibrate, therefore there are no laryngeal obstacles to air outflow) were decreased compared to other sustained phonemes, although still within normal limits; this may occur possibly due to lower respiratory control.^3^, ^4^, ^6^, ^7^, ^8^, ^15^, ^27^, ^29^

The statistical analysis showed that /a/, /s/, and /z/ MPT measurements had a significant positive correlation; in other words, as the mean /a/ MPT measurement (vowel) of the full sample increased, so did the /s/ and /z/ MPT measurements (fricatives).

The positive correlation was higher between the /a/ MPT and the /z/ MPT measurement; the mean single values of their MPTs were close, with an /a/ value higher than a /z/ value. These findings are not in agreement with those in the literature, which states that the sustained fricative /z/ has two obstruction points to the passage of air (glottic and articulatory points). In theory, air outflow would be more obstructed compared to the /a/ MPT, which would yield increased sustained values for /z/ MPT measurements.^3^, ^4^, ^6^, ^11^, ^15^

A positive correlation was seen between MPT /z/ and /s/ MPT and /a/ and /s/ MPT measurements, similar to /a/ and /z/ MPT measurements. As the /z/ MPT measurements increased, so did the /s/ MPT measurements; a similar finding was seen between the /a/ MPT and the /s/ MPT measurements. The /s/ MPT measurements were slightly lower than the /z/ MPT measurements, with a larger difference compared to the /a/ MPT measurements (/a/ MPT > /z/ MPT > /s/ MPT), in accordance with the literature. According to the literature,^4^, ^27^ the /z/ MPT and the /s/ MPT measurements should be close; the /z/ MPT measurement tends to be slightly higher (by up to 3 s) than the /s/ MPT in normal speakers, due to the association between glottic and articulatory control and the higher vocal fold closure quotient that takes place with the phoneme /z/. The difference between the /s/ and /z/ MPT measurements in our results was lower than the values found in the literature and in another study^30^ in which the /z/ MPT in its subjects was on average 2 s longer than the /s/ MPT. The explanation given was a laryngeal valve effect for the /z/ phoneme, as has also been described in other studies.^27^

Our conflicting findings with the literature may be explained as follows: the age four year means had a strong influence on the mean of the full sample; at this age, there is less control of the expiratory airflow,^3^, ^11^, ^15^, ^3^0 of the vocal fold neuromuscular control and of articulation, all of which affect mean /z/ MPT measurements.

The s/z ratio should be calculated in children, as it may indicate poor laryngeal function due to vocal fold injury.^31^ Some authors have stated that this measurement relates expiratory and laryngeal control.^3^, ^4^, ^6^, ^8^, ^11^, ^27^ The s/z ratio means in our results for all age groups were close to 1; comparing this results with those in the literature, only the age four year mean was slightly higher, although not significantly. A study of children aged seven to eleven years^32^ yielded results close to ours; in that study, the s/z ration was 1.02.

A study that also investigated the s/z ratio found that its mean for subjects aged between four and eleven years^15^ with no vocal fold injury was 1.06, which was higher than our findings. Researchers investigating the s/z ratio in children aged five, seven and nine years have found that the /s/ and /z/ MPT measurements increased gradually in proportion to the age, but that the s/z ratio remained stable even at different age groups.^24^ This finding is similar to our results, which showed that the /s/ and /z/ MPT means increased with age, but that the s/z ration did not vary significantly among the age groups.

In general, most of our results were in agreement with those in the literature, which reinforces similar Brazilian and international studies. Diverging results from the literature may stimulate further research that may increase our knowledge on this theme, providing additional data to support clinical evaluations in speech therapy, otorhinolaryngology and singing.

## CONCLUSION

Based on our purpose of adding to MPT measurements in children aged four to six years, of both sexes, we concluded that:

/a/ MPTs for ages four, five and six years were 5.77 s, 7.16 s and 10.32 s, all within normal limits as defined by Brazilian and international studies, but above proposed values in the Brazilian literature for these age groups;
/z/ /z/ MPTs for ages four, five and six years were 5.32 s, 7.30 s and 9.55 s, all within normal limits as defined in the literature, but higher than the results of Brazilian studies and lower than results found in some international papers for these age groups;
/s/ /s/ MPTs for ages four, five and six years were 4.73 s, 6.35 s and 8.62 s, all within normal limits as defined in the literature, but higher than the results of Brazilian studies and lower than those found in some international papers for these age groups;
/z/ /a/, /s/ and /z/ MPTs at age six years were significantly higher than the /a/ MPT at age four years. As age increased, /a/, /s/ and /z/ MPTs also increased significantly; the same was observed for the total means of the /a/, /s/ and /z/ MPTs. This shows that as the child develops physically, there is nervous and muscular maturation and increased neuromuscular control, as demonstrated in Brazilian and international studies. It was clear that MPT results at age four years were due to immaturity at this age;

the s/z ratios for ages four, five and six years were close to 1; this results for the full sample was 0.99. This is similar to the results in other studies and shows that this measurement should not be used singly, but in association with other measurements, as altered /s/ and /z/ MPTs may results in a normal s/z ratio, and therefore a false negative results.

These results need to be supported by similar studies with larger samples. This paper may, however, provide support for the evaluation of voice and MPT measurements in children.
